# Cortical Structure Abnormalities in Patients With Noise‐Induced Hearing Loss: A Surface‐Based Morphometry Study

**DOI:** 10.1002/brb3.70813

**Published:** 2025-09-10

**Authors:** Yunxin Li, Ranran Huang, Aijie Wang, Liping Wang, Minghui Lv, Xianghua Bao, Guowei Zhang

**Affiliations:** ^1^ Radiology Department Yantaishan Hospital Yantai Shandong China; ^2^ Occupational Department Yantaishan Hospital Yantai Shandong China

**Keywords:** cortical thickness, cortical volume, noise‐induced hearing loss, source‐based morphometry, surface area

## Abstract

**Objective:**

To investigate the characteristics of brain structures in patients with noise‐induced hearing loss (NIHL) using source‐based morphometry (SBM) and to evaluate the correlation between abnormal brain regions and clinical data.

**Methods:**

High‐resolution 3D T1 structural images were acquired from 81 patients with NIHL and 74 age‐ and education level‐matched healthy controls (HCs). The clinical data of all subjects were collected, including noise exposure time, monaural hearing threshold weighted values (MTWVs), Mini‐Mental State Examination (MMSE), and Hamilton Anxiety Scale (HAMA) scores. The FreeSurfer software was used to perform whole‐brain SBM analysis based on T1 images. The cortical morphological parameters included cortical thickness, surface area, and cortical volume. The correlations between abnormal cortical morphological changes in brain regions and noise exposure time, MTWV (superiority), and HAMA scores were further analyzed.

**Results:**

SBM results: NIHL patients had a greater cortical thickness in the left inferior parietal gyrus (IPG) and right superior parietal gyrus (SPG) compared with HCs (*t* = 2.916, *p* = 0.0474; *t *= 2.916, *p* = 0.0046); increased surface area and cortical volume were observed both in the left lateral occipital gyrus (LOG) in NIHL patients (*t* = 3.468, *p *= 0.0377; *t* = 3.091, *p* = 0.0002). Correlation analysis revealed that MTWV (superiority) was positively correlated with noise exposure time (*r* = 0.260, *p = *0.019), and HAMA scores were negatively correlated with noise exposure time (*r* = −0.297, *p *= 0.007). Further, thicker cortical thickness in the left IPG and right SPG and increased cortical volume in the left LOG were negatively correlated with MTWV (superiority) (*r* = −0.238, *p* *=* 0.032; *r* = −0.255, *p* *=* 0.022; *r* = −0.272, *p* *=* 0.014). Increased cortical volume in the left LOG had a positive correlation with HAMA scores (*r* = 0.172, *p* *=* 0.032).

**Conclusions:**

On the basis of the SBM method, we have discovered alterations in cortical morphology within the auditory–visual network regions such as the parietal and occipital lobes in NIHL patients. These findings suggest that auditory deprivation may initiate the restructuring of the auditory–visual cortex, providing new insights into the underlying mechanisms of brain activity abnormalities in NIHL patients. This study not only offers a new perspective on exploring brain structural anomalies associated with NIHL but also enhances our understanding of the neurobiology of NIHL.

## Graphical abstract

• To investigate the characteristics of brain structures in patients with noise‐induced hearing loss (NIHL) using source‐based morphometry (SBM).
• To evaluate the correlation between abnormal brain regions and clinical data.
• On the basis of the SBM method, complex cortical structural alterations in patients with NIHL predominantly involved the parietal and occipital lobes and other audiovisual network cortices, suggesting that auditory deprivation leads to remodeling of the audiovisual cortex brain structure, which helps to further explain the mechanism underlying abnormal brain activity in NIHL.
• Morphometric alterations in these specific regions may serve as promising markers for the monitoring and assessment of NIHL.

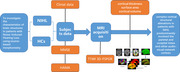

## 1 Introduction

Noise‐induced hearing loss (NIHL) is a form of bilateral symmetrical high‐frequency sensorineural hearing loss (SNHL) caused by long‐term unprotected or ineffective exposure to occupational noise during work loss (Massawe and Rahib 2023; Balk et al. 2023). NIHL is a complex occupational disease of the auditory system that further indirectly affects blood pressure, endocrine system, cardiovascular system, and even depression, which is harmful to occupational health and has a considerable negative impact on people's physical and mental health (Zhou et al. 2019; Kuleshova and Pankov 2021). Recently, due to industrial development, NIHL has become a more prevalent occupational disease worldwide (Liu, He, et al. 2024), and occupational health researchers worldwide are paying increasing attention to early warnings and comprehensive evaluation of occupational noise hazards.

Magnetic resonance imaging (MRI) is widely used to assess functional and structural changes in disease‐associated brain regions. Many studies have confirmed that patients with SNHL have abnormal brain structures and function (Cui et al. 2023; Giroud et al. 2021; Huang et al. 2020; Huang et al. 2023; Isler et al. 2021; Liu, Shi, et al. 2024; Neuschwander et al. 2023; Profant et al. 2013; Qu et al. 2020; Ranran et al. 2024; Siegbahn et al. 2024; Shiohama et al. 2019; Wang, Cui, et al. 2018); Shiohama et al. (2019) showed that the local cortical thickness of the left middle and left inferior occipital lobes was decreased in SNHL and may be related to visual cognitive dysfunction; Siegbahn et al. (2024) found that in patients with unilateral congenital conductive hearing impairment, the cortical volume of the primary auditory cortex was atrophies or changes in the asymmetric pattern; Neuschwander et al. (2023) showed that there were cortical morphological changes in age‐related hearing loss, mainly concentrated in the superior temporal gyrus and transverse temporal gyrus cortical volume and cortical thickness, and the cortical surface area did not change significantly. Zolkefley et al.’s (2024) study found that the abnormal microstructure integrity of white matter tracts in patients with NIHL involved the central auditory pathway, which may have a broad impact on brain function; Huang et al. (2024) also found that there were abnormal values of diffusion tensor imaging (DTI) parameters mainly in the inferior longitudinal fasciculus (ILF) under the white matter fiber tracts in the audiovisual cortex, suggesting that the white matter microstructure changes (myelin and axonal dysfunction) were related to brain structural reorganization after noise deprivation. Previous functional magnetic resonance (fMRI) studies revealed abnormal amplitude of low‐frequency fluctuation (ALFF) and regional homogeneity (ReHo) in the right angular gyrus (Huang et al. 2020; Yunxin et al. 2023). Static network analysis based on independent component analysis shows that there is an increase in functional network connections within and between networks for inferior parietal gyrus (IPG) (Ranran et al. 2024); effective quantitative functional connectivity based on Granger causality analysis (GCA) indicates a decreased connection from the left temporal transverse to the left precuneus (Aijie et al. 2024); further analysis revealed that the brain regions involved in these abnormal parameters were mainly concentrated in the related perceptual function brain regions with the audiovisual cortex as the hub. Luan et al. (2021) explored the GCA of NIHL rats based on Rs‐fMRI, which showed that the causal connection from the anterior limbic cortex to vision in noise‐exposed rats was significantly enhanced. Moreover, the small‐world network structure and network separation occurred in the functional connectivity of the whole brain, which provides important clues for understanding the cross‐modal functional remodeling caused by hearing loss after noise. Cross‐modal plasticity can occur as a result of reduced or abnormal sensory input (Glick and Sharma 2016), and these prior studies were all consistent with auditory deprivation leading to a cross‐modal plasticity mechanism in the cortex.

MRI‐technology‐based voxel‐based morphometry (VBM) and surface‐based morphometry (SBM) methods have allowed the quantitative analysis of changes in brain structure in the early stages of disease. VBM is a voxel‐based segmentation technology for brain structure processing that can quantitatively obtain brain gray matter and white matter information, detect subtle changes in brain structural volume and structure density, and accurately assess the changes in brain structure caused by different diseases. Some studies using VBM analysis have further identified the presence of brain microstructure changes in SNHL patients. For example, Cao and Guan (2020) analyzed patients with congenital hereditary hearing loss using VBM, finding that gray matter volume changes in cerebral cortex. Similarly, Zhang et al. (2023) also found that congenital severe SNHL patients exhibit gray matter density changes by VBM analysis. All of these studies suggest the presence of structural reorganization in SNHL patients. Cortical morphological parameters measured by SBM, including cortical thickness, cortical volume, and surface area, can simultaneously analyze the neuroanatomy of the gray matter structure from multiple dimensions at the millimeter level; however, VBM analysis cannot measure these changes (Tang et al. 2023). In recent years, SBM has been widely used in the investigation of Alzheimer's disease, epilepsy, Parkinson's disease, schizophrenia, and other diseases (Wu et al. 2021; Li et al. 2020; Wang et al. 2021; Li et al. 2019). For example, Li et al. (2021) used the SBM method to identify complex cortical structural alterations in patients with postpartum depression. Similarly, Ran et al. (2023) found that SBM and VBM analysis methods can detect brain regions with abnormal brain morphology in children and adolescents with frontal lobe epilepsy, whereas SBM can extract more abnormal brain regions than VBM.

Our previous study using VBM analysis identified differences in the gray matter volume of the left lateral occipital temporal gyrus, anterior cingulate gyrus, bilateral angular gyrus, and precuneus in patients with NIHL (Wang, Cui, et al. 2018). In recent years, SBM has been applied to the study of topics related to hearing (Qin et al. 2025; Kumar and Mishra 2018; Kumar et al. 2020). On the basis of this knowledge, in the present study, we applied the SBM brain morphological analysis method to explore the morphological characteristics of cerebral cortical microstructural changes in patients with NIHL to understand the brain structural changes and pathophysiological mechanisms associated with this condition.

## 2 Materials and Methods

### 2.1 Clinical Data

This study recruited 81 patients with NIHL diagnosed by occupational doctors from February 2014 to December 2022 following the Chinese national occupational hygiene standards (China Standards Press 2014). In addition, 74 age‐ and education‐matched healthy controls (HCs) were also enrolled, and the superiority monaural hearing threshold weighted value (MTWV) was within the normal range of 0–25 dB. All subjects completed the Mini‐Mental State Examination (MMSE), which is scored out of a total of 30 points; a score of 27–30 was normal, and a score <27 indicates cognitive dysfunction. All subjects with cognitive dysfunction were excluded. All patients also completed assessment of the Hamilton Anxiety Scale (HAMA) scores. Clinical data (age, educational level, noise exposure time, and MTWV (superiority: better superiority of both ears pure‐tone audiometry test)) were also collected.

According to China's national occupational criteria for NIHL (China Standards Press 2014), a diagnosis is made if the binaural high‐frequency (3000, 4000, and 6000 Hz) average hearing threshold exceeds 40 dB. NIHL is classified according to the weighted values of good whisper frequency (500, 1000, and 2000 Hz) and high‐frequency (4000 Hz) thresholds as follows: (A) mild NIHL: 26–40 dB; (B) moderate NIHL: 41–55 dB; (C) severe NIHL: ≥56 dB.

The inclusion criteria were as follows (Ranran et al. 2024): adult males aged 35–60 years; right‐handed; Han Chinese ethnicity; primary school to university level of education; normal mental state with no neuropsychiatric diseases, no systemic diseases, or other factors that may affect brain structure or function (ear MR examinations were normal); and not taking sedatives or central nervous system depressants. The exclusion criteria were as follows: illiterate, noncooperative, or contraindications for MRI. Before the start of the experiment, all subjects were informed of the purpose, procedures, and related contraindications.

### 2.2 MRI Acquisition

High‐resolution 3D T1 structural images were obtained using a GE Discovery MR 750 3.0T scanner (GE Healthcare) equipped with an eight‐channel head coil. Earplugs and headphones were used to alleviate noise during scanning. All subjects were required to lie flat, be quiet, close their eyes, and ensure emotional stability during scanning. The imaging parameters were as follows: TR = 6.9 ms, TE = 3.4 ms, slice thickness = 1 mm, no gap, FOV = 256 × 256 mm^2^, matrix = 256 × 256, NEX = 1 and flip angle = 12, with a scanning time of 4 min and 33 s.

### 2.3 Surface‐Based Morphometric Analysis

The FreeSurfer Image Analysis Suite (http://surfer.nmr.mgh.harvard.edu, V7.2.0) was used to analyze the structural T1 images. FreeSurfer has been widely used in the study of SBM (Dale et al. 1999; Fischl and Dale 2000).

**FIGURE 1:**
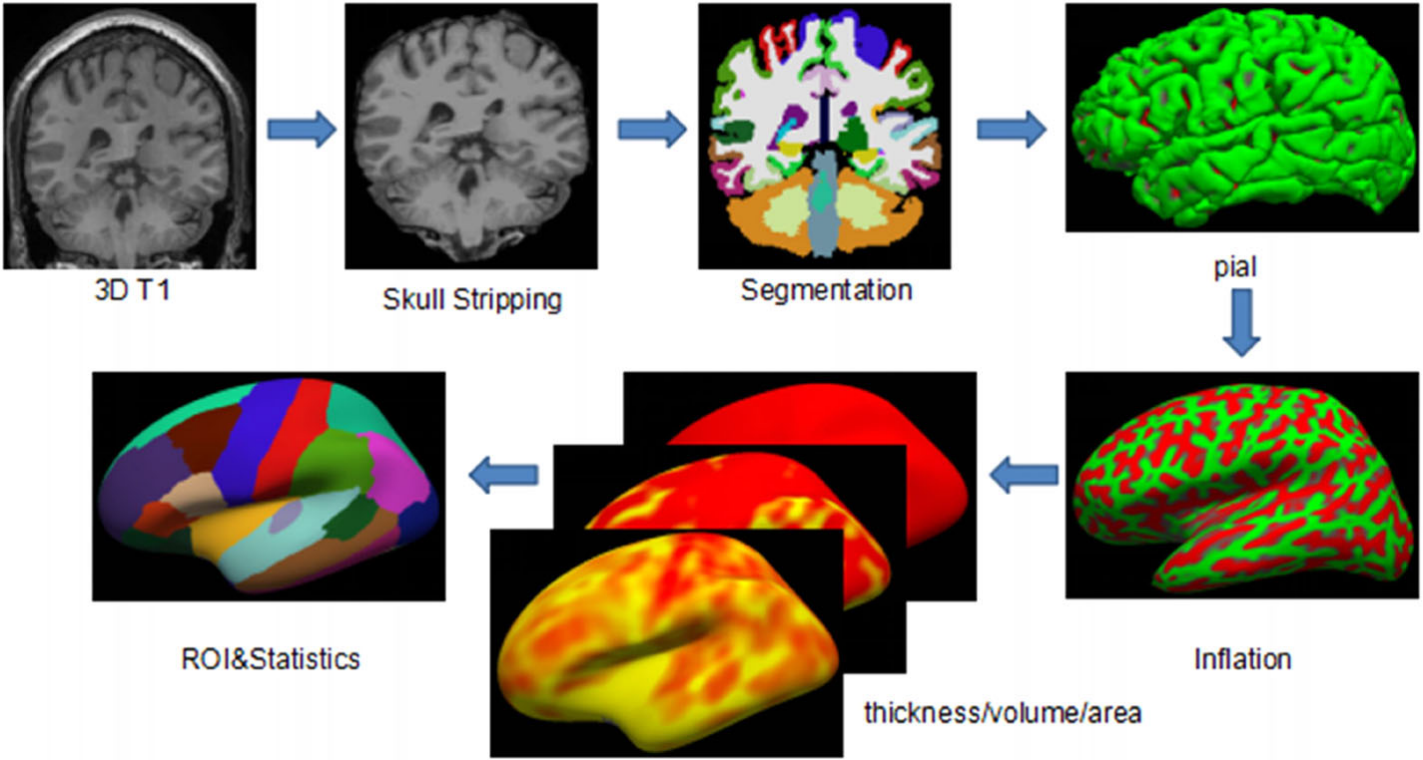
FreeSurfer whole‐brain analysis of the SBM flowchart.

Preprocessing is a crucial step in modern neuroimaging data analysis, which directly affects the quality of subsequent analysis and the reliability of the results. As a core component of preprocessing, feature reduction techniques not only help to improve computational efficiency but also enhance data interpretability. The process of data processing using FreeSurfer (shown in Figure 1) is as follows:

1. Correction: First, the magnetic field inhomogeneity correction is performed to reduce the signal distortion caused by the change of magnetic field intensity. Then motion correction was performed to eliminate disturbances such as head movement.
2. Standardization: Talairach transformation was used to match the anatomical structure of individual brain images with the standard space to achieve spatial standardization of data.
3. Tissue segmentation: Non‐brain tissues, such as scalp and skull, were removed, and gray matter, white matter, and cerebrospinal fluid in the image were segmented.
4. Montreal Neurological Institute (MNI) coordinated space registration: Each subject's image was registered to the MNI coordinate space.
5. Cortical skeleton reconstruction: The cortical surface was generated from three‐dimensional brain structure data, and the cortical skeleton was reconstructed.
6. Spherical extension and spatial registration: The cortical surface was extended to the spherical surface, and the spatial registration was performed.
7. Cortical partition: The Desikan–Killiany method was used to divide the cortex into 68 brain regions.
8. Morphological calculation and statistics: Each region of interest (ROI) was smoothed by 10 mm FWHM, and the average morphological parameters of each ROI were calculated and statistically analyzed.

### 2.4 Statistical Analysis

Statistical analyses were performed using Social Sciences software (SPSS Statistics 25.0). A two‐sample *t*‐test was used to assess potential differences in age, education level, HAMA score, and MMSE score (*p *< 0.05, indicating statistical significance) between the NIHL and HC groups.

Using the General Linear Model (GLM) tool in FreeSurfer to analyze differences in cortical thickness, surface area, and cortical volume between the NIHL group and the HC group, Monte Carlo simulation and Gaussian Random Field (GRF) correction were used for multiple comparison correction (Hagler et al. 2006). A vertex‐level threshold of *p *< 0.001 (two‐tailed) was set to identify clusters that may exhibit differences, and clusters were reported only if they met a cluster probability of at least *p *< 0.05 (two‐tailed).

According to the Shapiro–Wilk test, the cortical morphological parameters and clinical data follow a normal distribution. Pearson's correlation was used to analyze the correlation between noise exposure time, MTWV (superiority), and HAMA score, and the correlation between cortical morphological parameters with significant abnormalities (cortical thickness, cortical volume, and surface area) and noise exposure time, MTWV (superiority), and HAMA score. *p *< 0.05 was considered statistically significant.

## 3 Results

### 3.1 General Information

The demographic characteristics of the two groups were as follows: HC group (*n* = 74) mean age: 47.18 ± 7.5 years, education level: 11.14 ± 2.43 years; NIHL group (*n* = 81) mean age: 45.54 ± 6.62 years, education level: 10.58 ± 2.10 years. No significant differences were observed in terms of age, years of education, or MMSE scores between the two groups (*p *> 0.05); however, there was a statistically significant difference in HAMA scores (*p *< 0.05) (Table 1).

**TABLE 1:** Summary of the clinical characteristics of the study subjects (mean ± standard deviation).

	HCs (*n* = 74)	NIHL (*n* = 81)	*t* value	*p* value
Age (years)	47.18 ± 7.5	45.54 ± 6.62	1.439	0.152
Education level (years)	11.14 ± 2.43	10.58 ± 2.10	1.524	0.130
MMSE score	28.86 ± 1.08	29.00 ± 0.00	−1.130	0.260
HAMA score	3.80 ± 1.05	7.36 ± 3.82	−7.751	<0.001*
Noise exposure time (years)	—	15.67 ± 7.71	—	—
MTWV (superiority)(dB)	—	34.29 ± 6.21	—	—

*Note*: Significance was evaluated using a two‐sample *t*‐test between groups.

Abbreviations: HAMA, Hamilton Anxiety Scale; MMSE, Mini‐Mental State Examination; MTWV, monaural hearing threshold values; NIHL, noise‐induced hearing loss.

*Represented significant differences (*p *< 0.05).

### 3.2 Morphological Parameter Analysis

**FIGURE 2:**
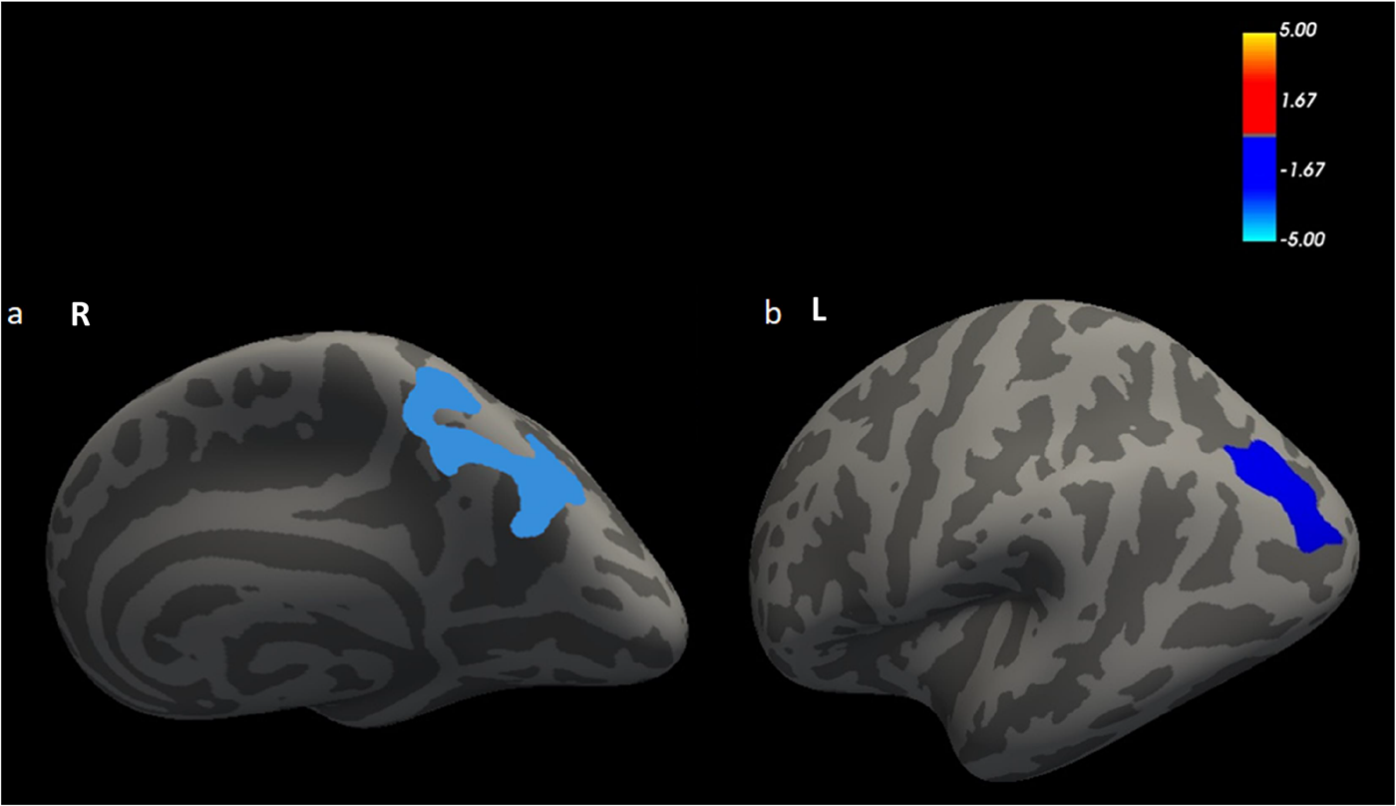
FreeSurfer whole‐brain analysis of cerebral cortical thickness.The cerebral surfaces exhibited differences in the cortical thickness of the right superior parietal gyrus (SPG)(a) and left inferior parietal gyrus (IPG)(b)between the two groups; colored regions indicate significant differences in cortical thickness between the two groups. Warm colors indicate HCs > NIHL; Cool colors indicate HCs < NIHL. The color bar shows the values as log10 (p value).

**FIGURE 3:**
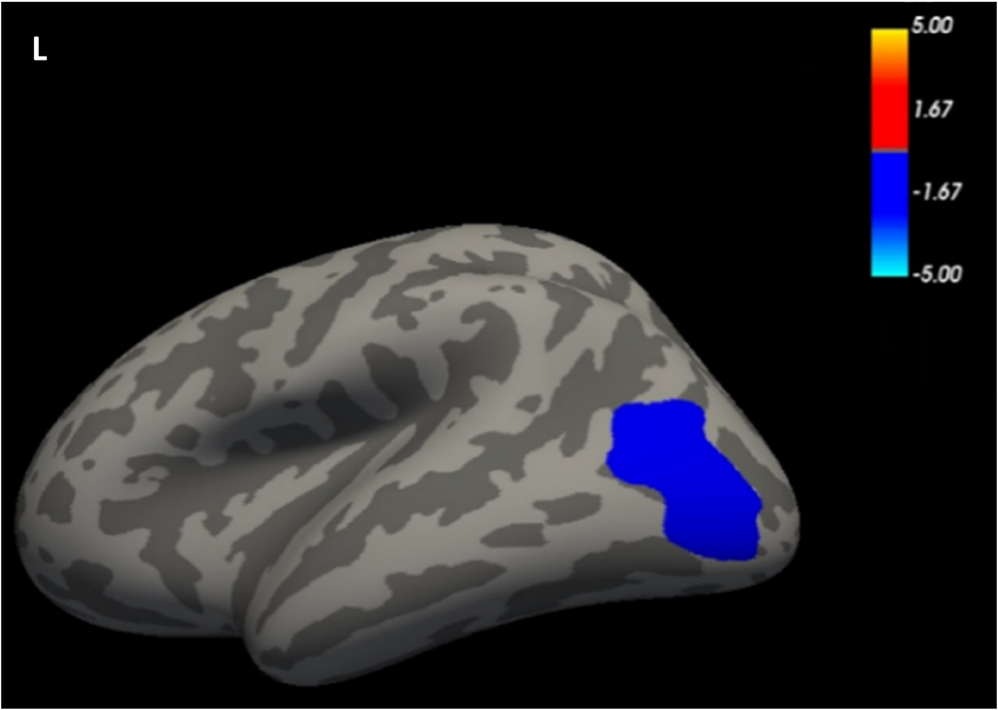
FreeSurfer whole‐brain analysis of surface area.

**FIGURE 4:**
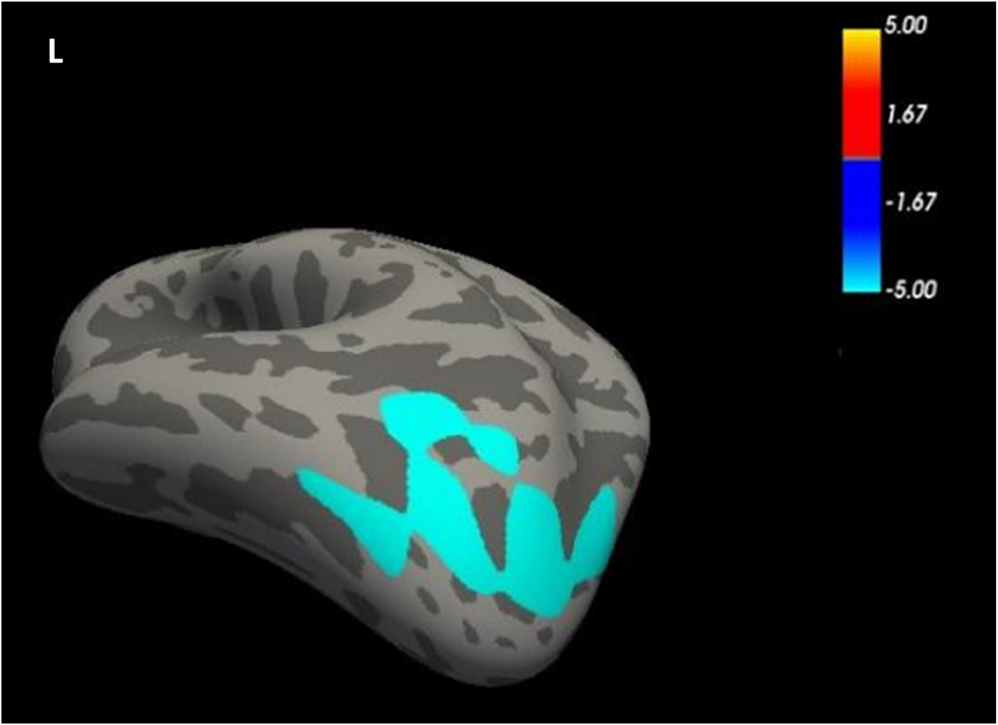
FreeSurfer whole‐brain analysis of cortical volume.

Compared to the HC group, the NIHL group showed increased cortical thickness in both the left IPG (*t *= 2.916, *p *= 0.0474) and right superior parietal gyrus (SPG) (*t *= 2.956, *p *= 0.0046) (*p* < 0.05, GRF‐corrected; Table 2, Figure 2a,b). Significantly increased surface area and cortical volume were further found in the left lateral occipital gyrus (LOG) in the NIHL group compared to the HCs group (*t* = 3.468, *p *= 0.0377; *t* = 3.091, *p *= 0.0002) (*p* < 0.05, GRF‐corrected; Table 2, Figures 3 and 4). Table 2 shows a summary of the different brain regions and specific information of the two groups.

**TABLE 2:** Regions with significant differences in morphological parameters between noise‐induced hearing loss (NIHL) patients and healthy controls (HCs).

Brain region	SBM parameter	Hemisphere	MNI peak point coordinates			*t* value	*p* value
			*X*	*Y*	*Z*		
Superior parietal gyrus (SPG)	Cortical thickness	R	17.6	−76.2	40.2	2.956	0.0046
Inferior parietal gyrus (IPG)	Cortical thickness	L	−33.5	−78.2	19.3	2.916	0.0474
Lateral occipital gyrus (LOG)	Surface area	L	−41.8	−75.4	−4.3	3.468	0.0337
Lateral occipital gyrus (LOG)	Cortical volume	L	−40.8	−83.8	3.1	3.091	0.0002

*Note: p* < 0.05, GRF‐corrected.

Abbreviations: HCs, healthy controls; L, left; MNI, Montreal Neurological Institute; NIHL, noise‐induced hearing loss; R, right; SBM, source‐based morphometry.

The cerebral surfaces exhibited differences in the cortical thickness of the SPG (a) and IPG (b) between the two groups; colored regions indicate significant differences in cortical thickness between the two groups. Warm colors indicate HCs > NIHL; cool colors indicate HCs < NIHL. The color bar shows the values as log10 (*p* value).

The cerebral surfaces show differences in the surface area of the LOG between the two groups. Warm colors indicate HCs > NIHL; cool colors indicate HCs < NIHL. The value of the color bar is log10 (*p* value).

The cerebral surfaces show differences in the cortical volume of the LOG between the two groups. Warm colors indicate HCs > NIHL; cool colors indicate HCs < NIHL. The value of the color bar is log10 (*p* value).

### 3.3 Correlation Analysis

**FIGURE 5:**
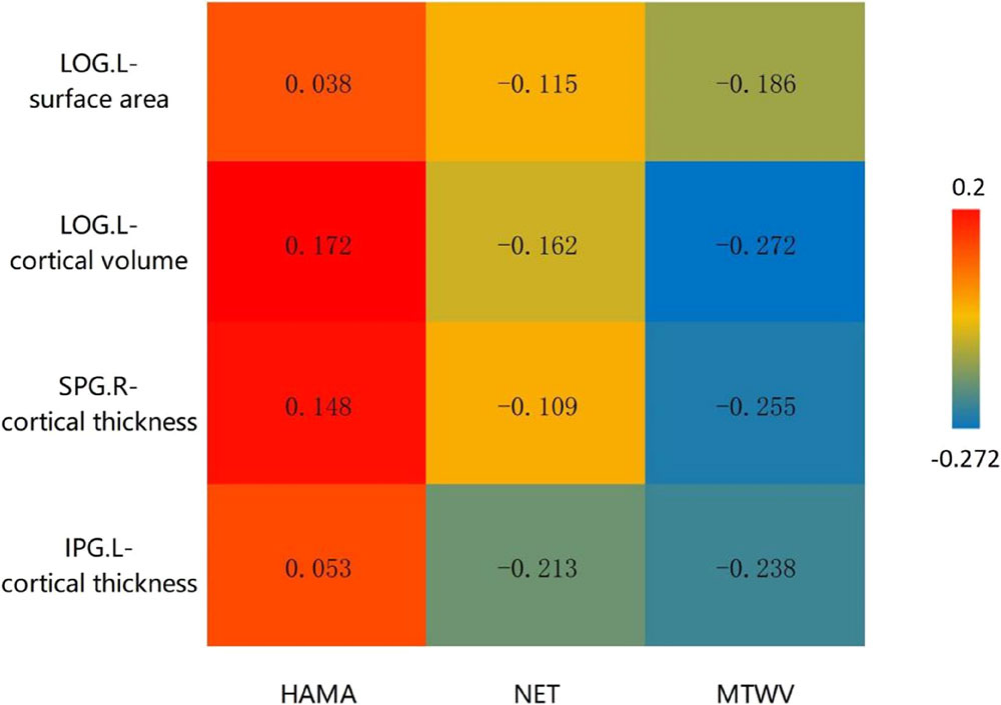
Correlation between the clinical data and morphological parameters. Values are shown as *r* values. HAMA, Hamilton Anxiety Scale; IPG, inferior parietal gyrus; LOG, lateral occipital gyrus; MTWV, monaural hearing threshold weighted values; NET, noise exposure time; SPG, superior parietal gyrus.

MTWV (superiority) was positively correlated with noise exposure time (*r* = 0.260, *p = *0.019). The HAMA scores were negatively correlated with noise exposure time (*r* = −0.297, *p *= 0.007). Increased cortical thickness in the left IPG and right SPG were negatively correlated with the MTWV (superiority) (*r* = −0.238, *p = *0.032; *r *= −0.255, *p = *0.022, Table 3, Figure 5). Increased cortical volume in the left LOG was negatively correlated with the MTWV (superiority) (*r* = −0.272, *p = *0.014, Table 3, Figure 5). Increased cortical volume in the left LOG was also positively correlated with HAMA scores (*r* = 0.172, *p = *0.032; Table 3, Figure 5). Overall, the results showed that there was no significant correlation between the morphological parameters and noise exposure time (*p *> 0.05).

**TABLE 3:** Correlation between the clinical data and morphological parameters.

Clinical data	Significant differences morphological parameters	*r* value	*p* value
MTWV (superiority (dB))	LOG.L.surface area	−0.183	>0.05
	LOG.L.cortical volume	−0.272	0.014*
	SPG.R.cortical thickness	−0.255	0.022*
	IPG.L.cortical thickness	−0.238	0.032*
Noise exposure time (years)	LOG.L.surface area	−0.115	>0.05
	LOG.L.cortical volume	−0.162	>0.05
	SPG.R.cortical thickness	−0.109	>0.05
	IPG.L.cortical thickness	−0.213	>0.05
HAMA score	LOG.L.surface area	0.038	>0.05
	LOG.L.cortical volume	0.172	0.032*
	SPG.R.cortical thickness	0.148	>0.05
	IPG.L.cortical thickness	0.053	>0.05

Abbreviations: HAMA, Hamilton Anxiety Scale; IPG, inferior parietal gyrus; LOG, lateral occipital gyrus; MTWV, monaural hearing threshold weighted values; NET, noise exposure time; SPG, superior parietal gyrus.

*Represents that there is a significant correlation between clinical data and significant differences in morphological parameters, *p *< 0.05.

## 4 Discussion

In recent years, the neurobiological mechanisms underlying NIHL have been widely investigated. However, to the best of our knowledge, no imaging studies have yet examined the cortical morphometric alterations in patients with NIHL. In the present study, FreeSurfer‐SBM was used to quantitatively analyze the changes in brain structural characteristics in NIHL patients, revealing differences in the cortical structure in multiple brain regions. This study revealed differences in the cortical structure in several brain regions, including greater cortical thickness in the left IPG and right SPG and increased surface area and cortical volume in the left LOG region. These results suggest that SBM can identify cortical structural changes related to neurological functional changes in patients with NIHL and provide new insights into the neuropathological mechanisms of NIHL.

Some studies have shown that SBM detects more abnormal brain regions than VBM (Abuaf et al. 2022; Chen et al. 2022), further speculating that the reason for the difference between the two methods may be related to the fact that VBM uses less prior information analysis, whereas SBM requires more prior information analysis. Conversely, some scholars have proposed that VBM has low sensitivity to brain structure in brain tissue segmentation, and image smoothing may also reduce its sensitivity and accuracy in detecting structural changes (Ziaei et al. 2022; Ioannidis 2011). Cortical morphological parameters measured using SBM include cortical thickness, cortical volume, and surface area. Cortical thickness reflects the number and density of cells in a certain area, whereas surface area reflects the number of columnar arrangements of neurons in the cerebral cortex. Some studies have shown that the cortical thickness and surface area of the brain are genetically and developmentally independent (Docherty et al. 2015; Jha et al. 2018; la Fougère et al. 2010). Cortical volume is determined by cortical thickness and surface area, which are subject to different evolutionary, genetic, and cellular processes, showing unique changes at different life stages (Docherty et al. 2015). Therefore, the SBM may reflect subtle structural changes in the cortex better than VBM.

### 4.1 Cortical Thickness

Structural changes in the brain and functional remodeling processes in patients with hearing loss have been inconsistently reported (Siegbahn et al. 2024; Liu, Shi, et al. 2024; Shiohama et al. 2019; Qu et al. 2020; Huang et al. 2020; Ranran et al. 2024). On the basis of the SBM method, the NIHL group showed increased cortical thickness in the left IPG and right SPG compared with HCs. The IPG is located at the temporal–occipital–parietal junction and comprises the supramarginal gyrus and adjacent angular gyrus, a convergence area that receives input from the sensory, motor, and emotional systems (Binder and Desai 2011). The left IPG is associated with higher level language functions, including phonological processing, speech perception, and auditory–visual integration. Cao and Guan (2020) and Zhang et al. (2023) used VBM to analyze severe congenital SNHL, finding that the IPG gray matter volume increased, consistent with the abnormal brain areas in this study. These findings corroborate the cortical remodeling in the IPG, indicating the presence of morphological changes in the visual–auditory cortex in patients with NIHL, which is consistent with the mechanism of functional reorganization. The IPG belongs to the executive control network (ECN) (Sherman et al. 2014), which is responsible for the active management of brain tasks and can coordinate a variety of brain dysfunctions, such as working memory, adaptability to cognitive tasks, problem‐solving, and planning. The thickening of the left IPG in patients with NIHL may develop as a result of the internal functional compensation of the ECN, which is related to hearing loss and deprivation of patients, leading to the uncontrollable focus of patients on certain things for supplementation (Ranran et al. 2024). The prior study found that damage to the ILF pathway will lead to object recognition defects, involving aspects such as visual processing, visual processing tasks, language, and semantic functions. Huang et al. (2024) found that there were abnormal values of DTI parameters in the ILF, suggesting that the white matter microstructure changes were related to brain structural reorganization after noise deprivation. The IPG and ILF cooperate with each other in visual information processing, cognitive function, and language function to support the complex functions of the brain. Given the increased cortical thickness in these regions in NIHL patients, this may indicate compensatory mechanisms related to speech perception and auditory–visual integration difficulties.

The SPG is the cortical center of tactile and entity perception, which means that familiar objects can be recognized by touch. It further represents an important node in the dorsal attention network (DAN) (Vincent et al. 2008). In the present study, the NIHL group showed an increased cortical thickness in the SPG, which may have been caused by hearing loss. It has been speculated that stimulation obtained by the relevant auditory cortex is reduced, leading to an integrated disorder of auditory and visual language stimulation. Cortical thickening results in better coordination and adaptation to repair brain structure and function, which may be a compensatory mechanism, indicating that human brain functional networks have plasticity under different physiological conditions to adapt to the needs of survival and changes in the surrounding environment (Huang et al. 2020; Ranran et al. 2024). As such, increased cortical thickness in the left IPG and right SPG indicates that hearing deprivation leads to a compensatory enhancement of visual and language abilities.

In this study, our research results did not reveal any alterations in the cortical thickness of the auditory brain regions. This is consistent with the results of Zolkefley et al. (2024) study. The possible reason might be that our research subjects were postlingual deaf individuals. Their auditory pathways were relatively intact, and there were no organic lesions in the auditory system. NIHL is a protracted process. Patients gradually accept and adapt to the noisy environment, and their auditory pathways do not undergo changes. In the study conducted by Butler et al. (2017), a cat study discovered that the pattern of connectivity that supports normal hearing is retained in the deaf animals brain, which also supports our research results to some extent. In hearing loss research, we did not find other human studies showing no change in cortical thickness in the temporal gyrus.

### 4.2 Surface Area and Cortical Volume

Our analyses using the SBM method revealed that the NIHL group showed increased surface area and cortical volume in the left LOG compared to HCs. The LOG is located in the posterior part of the skull, plays an important role in object recognition, visuospatial coordination, and motion perception, and has close structural connections with other cortex (Palejwala et al. 2019; Watanabe et al. 2011). The occipital lobe is the visual center, whereas modulation of visual reactivity by auditory inputs has been demonstrated in infants, adults, and mice (Petro et al. 2017; Iurilli et al. 2012; Rockland and Ojima 2003). Further research has shown a direct connection between the primary visual and auditory areas (Finney et al. 2003). Previous studies have also shown that the primary auditory cortex of deaf people can modulate visual stimuli, whereas visual stimuli can activate the auditory cortex of deaf people, and visual activity has been observed in the auditory cortex of deaf people (Finney et al. 2003). Congenital deafness has also been found to enhance peripheral visual processing (Scott et al. 2014), suggesting that there is an inherent cortical connection between visual and auditory modes in healthy people. When the auditory center is damaged, its function may be enhanced by the activation of the visual center. Sensory interactions occur between the auditory and visual cortex. Shi et al. (2016) also found that the connection between the visual cortex (LOG) and motor cortex was enhanced in children with SNHL, showing that auditory deprivation can lead to a compensatory increase in surface area and cortical volume in the left LOG, which is consistent with the mechanism of brain structural remodeling. Cao and Guan (2020) found that the cortical volume of the left superior occipital gyrus and left middle occipital gyrus in severe congenital SNHL patients was higher than that in controls, and this is partially consistent with the results of our study. Some studies have shown that there are interconnected pathways in different brain regions (Falchier et al. 2002), and more studies have suggested that the auditory center of deaf patients can be activated by a variety of tasks (such as vision), and there is cross‐area cortical deprivation (Finney et al. 2001; MacSweeney et al. 2001), which also provides a reasonable explanation for our research results. The reason may be related to the compensation mechanism formed by the increase of visual information after auditory deprivation, and there may be cross‐region cortical deprivation. However, Shiohama et al. (2019) found that the left LOG cortical thickness, surface area, and cortical volume of patients with moderate‐to‐severe infant SNHL were lower than those of a normal control group, which could be related to the long‐standing extremely low auditory inputs from the prelingual period. The opposing results identified in our study may be due to the fact that our subjects belong to postlingual deafness, whereas NIHL is a relatively long process, associated with slow weakening of the auditory system, hearing loss, and compensatory thickening of the visual center.

### 4.3 Clinical Correlation

Clinical correlations showed that the MTWV (superiority) was positively correlated with noise exposure time, confirming that long‐term noise exposure causes hearing loss, consistent with the mechanism of damage of NIHL (Fettiplace 2017).

HAMA scores were negatively correlated with noise exposure time in the NIHL group, and this may be because as the length of deafness is extended, patients show an increased psychological identity related to the state of deafness, whereas psychological adaptability gradually increases, resulting in the psychological suggestion of “coexistence” with deafness (Qi et al. 2015).

On the basis of SBM analysis, the results of related studies within the NIHL group showed that cortical morphological parameters (cortical thickness and cortical volume) were negatively correlated with MTWV, indicating that the NIHL group adapted to the noisy environment and deafness, which is consistent with the Ranran et al. (2024) observed reduction in brain microstructure. The clinical mechanism confirms that noise exposure is related to the MTWV (superiority), so it is speculated that the related changes in SBM may be related to the noise‐deafness environment, but it does not reach the statistical significance level.

In this study, the cortical volume in the left LOG was positively correlated with HAMA scores. Some studies suggest that anxiety levels are positively correlated with the structure and function of the occipital lobe (Brühl et al. 2014, Wang, Cheng, et al. 2018), which supports our results.

### 4.4 Limitations

This study has several limitations. First, there were no female patients, as we only enrolled male patients, meaning that the interpretation of the results was relatively limited. In the future, the sample size will be expanded to expand upon the research and discussion (including left‐handedness and racial differences). Another limitation lies in the fact that our current study focused on brain structural changes and did not combine it with state fMRI. In the future, we will conduct further research and incorporate measures such as diffusion MRI connectivity, as well as audiometric assessments.

## 5 Conclusions

In this study, using the SBM method, we found that NIHL patients have impaired hearing, whereas long‐term noise exposure triggers surface morphology changes in specific cortical areas, mainly involving the left IPG, right SPG, and left LOG, and other audiovisual network cortex. The temporal lobe, parietal lobe, and occipital lobe together constitute a complex and closely connected audiovisual network. The cross‐modal plasticity caused by auditory deprivation may lead to structural remodeling of the audiovisual cortex, which is helpful to further explain the mechanism of abnormal brain activity in NIHL. This study explored the brain abnormalities of NIHL from the perspective of structural imaging and provided neuroimaging basis for the subsequent diagnosis and treatment of patients with NIHL. Supporting Information

## Author Contributions

**Yunxin Li**: data curation, investigation, methodology, writing–original draft. **Ranran Huang**: data curation, writing–review and editing. **Aijie Wang**: data curation, writing–review and editing. **Liping Wang**: data curation. **Minghui Lv**: data curation. **Xianghua Bao**: data curation. **Guowei Zhang**: formal analysis, supervision.

## Ethics Statement

This study protocol was approved by the Yantaishan Hospital Ethics Committee.

## Consent

Informed consent includes appropriate statements (2023014). All participants provided written informed consent in accordance with the Declaration of Helsinki.

## Conflicts of Interest

The authors declare no conflicts of interest.

## Peer Review

The peer review history for this article is available at https://publons.com/publon/10.1002/brb3.70813.

## Supporting information




**Supplementary Materials**: brb370813‐sup‐0001‐SuppMat.pdf

## Data Availability

The data that support the findings of this study are available on request from the corresponding author. The data are not publicly available due to privacy or ethical restrictions. Code sharing: The recon‐all command in FreeSurfer is used for data preprocessing. The command format is as follows: “recon‐all ‐s <subname> ‐all‐qcache.” Details from: https://surfer.nmr.mgh.harvard.edu/fswiki/recon‐all. Statistical analysis using FreeSurfer is conducted through its graphical user interface by entering the “qdec” command in the terminal, which opens a new interface for manual statistical analysis operations. Details from: https://surfer.nmr.mgh.harvard.edu/fswiki/FsTutorial/QdecGroupAnalysisV6.0.
