# Supplementary material for: Cortical Structure Abnormalities in Patients With Noise‐Induced Hearing Loss: A Surface‐Based Morphometry Study
Source: Brain Behav. 2025 Sep 10;15(9):e70813. doi: 10.1002/brb3.70813 (PMC12422029; doi:10.1002/brb3.70813)
Supplement: Supplementary file 1 — Supplementary Materials: brb370813‐sup‐0001‐SuppMat.pdf [file BRB3-15-e70813-s001.pdf]

ICS 13.100
C 60

GBZ

中华人民共和国国家职业卫生标准

GBZ 49—2014
代替 GBZ 49—2007

职业性噪声聋的诊断

Diagnosis of occupational noise-induced deafness

2014-10-13发布

2015-03-01实施

中华人民共和国
国家卫生和计划生育委员会 发布

前　言

根据《中华人民共和国职业病防治法》制定本标准。

本标准第5章为推荐性的，其余为强制性的。

本标准按照GB/T 1.1—2009给出的规则起草。

本标准代替GBZ 49—2007《职业性噪声聋诊断标准》，与GBZ 49—2007相比，主要技术变化如下：

——取消了观察对象；

——将4 000 Hz听阈值纳入诊断分级指标中，进行加权计算；

——将双耳高频(3 000 Hz、4 000 Hz、6 000 Hz)平均听阈≥40 dB列为诊断职业性噪声聋的前提条件。

本标准负责起草单位：同济大学附属上海市肺科医院(上海市职业病医院)、上海市疾病预防控制中心。

本标准参与起草单位：北京市疾病预防控制中心、广东省职业病防治院、江苏省疾病预防控制中心、浙江省疾病预防控制中心、深圳市宝安区西乡卫生监督所。

本标准主要起草人：张巡淼、章敏华、王建新、阮艳君、张巧耘、杨爱初、江志荣、马谷丰、陈嘉斌、袁伟民、谢兰兰、顾明华。

本标准所代替标准的历次版本发布情况为：

——GB 16152—1996；

——GBZ 49—2002；

——GBZ 49—2007。

职业性噪声聋的诊断

1 范围

本标准规定了职业性噪声聋的诊断原则、诊断分级及处理原则。

本标准适用于长期职业接触噪声所致听力下降的诊断及处理。

2 规范性引用文件

下列文件对于本文件的应用是必不可少的。凡是注日期的引用文件，仅注日期的版本适用于本文件。凡是不注日期的引用文件，其最新版本(包括所有的修改单)适用于本文件。

GBZ 2.2 工作场所有害因素职业接触限值 第2部分：物理因素

GB/T 4854.1 声学 校准测听设备的基准零级 第1部分：压耳式耳机纯音基准等效阈声压级

GB/T 7341.1 电声学 测听设备 第1部分：纯音听力计

GB/T 7582 声学 听阈与年龄关系的统计分布

GB/T 7583 声学 纯音气导听阈测定 听力保护用

GB/T 16180 劳动能力鉴定 职工工伤与职业病致残等级

GB/T 16403 声学 测听方法 纯音气导和骨导听阈基本测听法

3 诊断原则

根据连续3年以上职业性噪声作业史，出现渐近性听力下降、耳鸣等症状，纯音测听为感音神经性聋，结合职业健康监护资料和现场职业卫生学调查，进行综合分析，排除其他原因所致听觉损害，方可诊断。

4 诊断分级

符合双耳高频(3 000 Hz、4 000 Hz、6 000 Hz)平均听阈≥40 dB者，根据较好耳语频(500 Hz、1 000 Hz、2 000 Hz)和高频4 000 Hz听阈加权值进行诊断和诊断分级：

a) 轻度噪声聋：26 dB～40 dB；

b) 中度噪声聋：41 dB～55 dB；

c) 重度噪声聋：≥56 dB。

5 处理原则

5.1 噪声聋患者均应调离噪声工作场所。

5.2 对噪声敏感者(上岗前职业健康体检纯音听力检查各频率听力损失均≤25 dB，但噪声作业1年之内，高频段3 000 Hz、4 000 Hz、6 000 Hz中任一耳，任一频率听阈≥65 dB)应调离噪声作业场所。

5.3 对话障碍者可配戴助听器。

5.4 如需劳动能力鉴定，按GB/T 16180处理。

6 正确使用本标准的说明

参见附录A。

附　录　A
（资料性附录）
正确使用本标准的说明

A.1　职业暴露于噪声作业引起听力损失的临床特点为早期以高频听力下降为主，可逐渐累及语频，导致感音神经性聋。本标准中的“噪声作业”指工作场所噪声强度超过“工作场所有害因素职业接触限值”的作业，即 8 h 等效声级（A 计权）≥85 dB（见 GBZ 2.2）。

A.2　职业性噪声聋的听力评定以纯音听阈测试结果为依据，纯音听阈各频率重复性测试结果阈值偏差应≤10 dB，听力损失应符合噪声性听力损伤的特点。为排除暂时性听力阈移的影响，应将受试者脱离噪声环境 48 h 后作为测定听力的筛选时间。若筛选测听结果已达噪声聋水平，应进行复查，复查时间定为脱离噪声环境后一周。

A.3　听力计应符合 GB/T 7341.1 的要求，并按 GB/T 4854.1 进行校准。

A.4　纯音听力检查时若受检者在听力计最大声输出值仍无反应，以最大声输出值计算。

A.5　纯音听力检查结果应按 GB/T 7582 进行年龄性别修正（见表 A.1）。

表 A.1　耳科正常人随年龄增长的听阈阈移偏差中值

年龄<br>岁	纯音气导听阈频率<br>Hz											
	500		1 000		2 000		3 000		4 000		6 000	
	男	女	男	女	男	女	男	女	男	女	男	女
20～29	0	0	0	0	0	0	0	0	0	0	0	0
30～39	1	1	1	1	1	1	2	1	2	1	3	2
40～49	2	2	2	2	3	3	6	4	8	4	9	6
50～59	4	4	4	4	7	6	12	8	16	9	18	12
60～69	6	6	7	7	12	11	20	13	28	16	32	21
70～	9	9	11	11	19	16	31	20	43	24	49	32

A.6　当一侧耳为混合性聋，若骨导听阈符合职业性噪声聋的特点，可按该耳骨导听阈进行诊断评定。若骨导听阈不符合职业性噪声聋的特点，应以对侧耳的纯音听阈进行诊断评定。

A.7　若双耳为混合性聋，骨导听阈符合职业性噪声聋的特点，可按骨导听阈进行诊断评定。

A.8　语言频率听力损失大于等于高频听力损失，不应诊断职业性噪声聋。

A.9　纯音听力测试结果显示听力曲线为水平样或近似直线、对纯音听力检查结果的真实性有怀疑，或纯音听力测试不配合，或语言频率听力损失超过中度噪声聋以上，应进行客观听力检查，如：听觉脑干诱发电位测试、40 Hz 听觉诱发电位测试、声阻抗声反射阈测试、耳声发射测试、多频稳态听觉电位等检查，以排除伪聋和夸大性听力损失的可能。

A.10　进行职业性噪声聋诊断，可按照以下步骤进行：

a)　耳科常规检查；

b)　至少进行 3 次纯音听力检查（纯音听阈测试按 GB/T 7583 和 GB/T 16403 规定进行），两次检查间隔时间至少 3 d，而且各频率听阈偏差应≤10 dB；诊断评定分级时应以每一频率 3 次中最小阈值进行计算。

c) 对纯音听力检查结果按 GB/T 7582 进行年龄性别修正(见表 A.1);

d) 进行鉴别诊断,应排除的其他致聋原因主要包括:伪聋、夸大性听力损失、药物(链霉素、庆大霉素、卡那霉素等)中毒性聋、外伤性聋、传染病(流行性脑脊髓膜炎、腮腺炎、麻疹等)性聋、家族性聋、梅尼埃病、突发性聋、各种中耳疾患及听神经瘤、听神经病等;

e) 符合职业性噪声聋听力损失特点者,计算双耳高频平均听阈(BHFTA),见式(A.1),双耳高频平均听阈≥40 dB 者,分别计算单耳平均听阈加权值(MTMV),以较好耳听阈加权值进行噪声聋诊断分级,见式(A.2);

f) 双耳高频平均听阈及单耳听阈加权值的计算(结果按四舍五入修约至整数),见式(A.1)和式(A.2)。

$$BHFTA = \frac{HL_{\mathrm{L}} + HL_{\mathrm{R}}}{6} \qquad \cdots\cdots (\mathrm{A.1})$$

式中:

$BHFTA$ ——双耳高频平均听阈,单位为分贝(dB);

HL_{L} ——左耳 3 000 Hz、4 000 Hz、6 000 Hz 听力级之和,单位为分贝(dB);

HL_{R} ——右耳 3 000 Hz、4 000 Hz、6 000 Hz 听力级之和,单位为分贝(dB)。

$$MTMV = \frac{HL_{500\ \mathrm{Hz}} + HL_{1\ 000\ \mathrm{Hz}} + HL_{2\ 000\ \mathrm{Hz}}}{3} \times 0.9 + HL_{4\ 000\ \mathrm{Hz}} \times 0.1 \qquad \cdots\cdots (\mathrm{A.2})$$

式中:

$MTWV$ ——单耳听阈加权值,单位为分贝(dB);

HL ——听力级,单位为分贝(dB)。
